# Electromagnetic Imaging for Breathing Monitoring

**DOI:** 10.3390/s24237722

**Published:** 2024-12-03

**Authors:** Ivan Vassilyev, Zhassulan Mendakulov

**Affiliations:** Special Design and Technology Bureau “Granit”, 292 Hussainov Street, Almaty 050060, Kazakhstan; iv@granit.kz

**Keywords:** medical imaging, image reconstruction algorithms, electromagnetic sensors for physiological monitoring, respiratory system, disease localization, microwave imaging techniques, electromagnetic medical imaging and sensing (EMIS)

## Abstract

The search for new non-invasive methods of investigating the functioning of human internal organs is an urgent task. One of these methods for assessing the functioning of the human respiratory system is electromagnetic sensing, which is based on a significant difference in the dielectric permittivity of muscle tissue and air. During breathing, when the lungs are filled with air, the dielectric permittivity of the lungs decreases, which leads to a change in the level of the electromagnetic signal passing through the body. The results of experiments on recording changes in the level of electromagnetic radiation passing through the human body performed on an experimental device consisting of eight transmitting and receiving antennas located on opposite sides of the chest have been presented in the article. The possibility of visualizing the measured “pulmonograms” in the form of dynamic two-dimensional images showing the process of filling various parts of the lungs with air has been demonstrated.

## 1. Introduction

The existing directions for the use of microwaves in medical tasks and the methods of obtaining images that are convenient for interpretation of the obtained results can be classified according to the object of investigation, the method of obtaining information, and the method of visualizing information.

In the article, the object of investigation is the human respiratory system. An investigation is carried out by the method of microwave spatial end-to-end scanning of the lungs during breathing by measuring the attenuation of the signals transmitted through the chest, followed by their conversion into pulmonograms. Visualization of the obtained information is carried out by converting individual pulmonograms measured in different areas of the lungs into dynamically changing two-dimensional color images.

The possibility of obtaining information about the human respiratory system by the chosen investigation method is based on the physical property that the dielectric permittivity of the lungs changes during breathing. A literature review revealed the fundamental differences between the chosen research method and the existing methods of investigating the human respiratory system using the microwave method. The presented method:

(1) is based on ***end-to-end microwave scanning*** of the object of investigation—the lungs and on measuring electromagnetic signal levels during breathing;
(2) does not depend on the specific values of the dielectric permittivity of the scanning medium;
(3) is aimed at obtaining information about ***dynamic changes*** in the object of investigation, which allows for seeing the air motion in the lungs;
(4) allows ***for scanning the entire volume of the lungs*** at almost the same time, which allows for carrying out the investigation on the task of air distribution in different areas of the lungs dynamically;
(5) uses ***noncoherent integration*** by synchronizing breathing cycles, which allows for increasing the signal-to-noise ratio and reliability of the measurement results.

To implement the human respiratory investigation system based on these methods in practice, a complex device was developed [1,2], and a series of numerous experiments were carried out on humans at the safe radiation power of the generator, namely from −30 dBm to −10 dBm at the frequency of 1220 MHz. 

The investigation of the human respiratory system using microwaves can be carried out in various ways [3,4,5,6,7,8,9,10,11,12,13]. 

In [3], the authors described a designed system for obtaining an image of the chest for human respiration monitoring. An array of 16 compact symmetrical dipole antennas with a low profile was installed in a belt fixed around the chest. During the measurement, one of the 16 antennas successively transmits the signal, and the rest receive it. During respiratory monitoring, S-parameters are measured. The Jacobian Weighted One-step Supervised Descent Method (SDM) is used to reconstruct the synthesized and measured data. As a result of tomography, the distribution of the dielectric properties of the chest is reconstructed. The measurements were carried out at frequencies of 433 MHz and 915 MHz. 

In [4], the authors proposed electromagnetic tomography based on microwave monitoring of the lung’s parameters (depth profiles of relative air and blood content) in the near zone by measuring variations in the shape of a scattered pulse for pulmonary and cardiovascular activity. The proposed tomography is based on solving the inverse scattering problem and allows for obtaining a quantitative distribution of the physical parameters of the lungs. The experimental results showed that the variations in the profile of the air content associated with breathing turned out to be quite small and could only be detected by the experimental device in some places of the chest. 

In [5], a device for diagnosing diseases of the bronchopulmonary system was proposed. The disadvantage of this system is that, in order to examine the patient, it is necessary to tightly press the receiving antenna applicators to the body and manually move the transmitting antenna of the applicator along the patient’s chest, which makes it difficult to obtain stable results. 

In [6], the authors described a method for diagnosing and monitoring pathological cardiopulmonary conditions, such as pulmonary edema by microwave penetrating radiation. Pulmonary edema causes changes in the electromagnetic characteristics in the lung tissues, which in turn causes changes in the reflected and penetrating radiation. The experiments were carried out using the applicator antenna. The disadvantage is the inability to study the entire volume of the lung’s dynamics. 

In review [7], the authors showed that measuring a penetrating electromagnetic wave provides more information than measuring a reflected wave. The scan was performed at a frequency of 915 MHz. In [8], the authors also compared reflection and transmission methods for use in detecting pathological changes in the lungs. The authors argue that the method based on a transmitting wave has the advantage that absorption does not depend on the path of propagation of the signal. It is noted in [9] that an electromagnetic signal with a wavelength shorter than 3 cm does not penetrate far into tissues, and it is recommended to use frequencies below 10 GHz. 

In [10], the author constructed a power values projection image of a microwave signal that passed through the human chest at a frequency of 2 GHz, moving one pair of antennas of the transmitter and receiver. The author notes that diffraction and interference effects make it difficult to reconstruct the internal structure of the human body. 

Works [11,12,13] are devoted to the use of radar to monitor human breathing using information about the movement of the chest. Radar data based on chest movement does not allow for a visualization of the internal structure of the lungs and has a different purpose. 

The COVID-19 pandemic stimulated the development of new methods for obtaining information about the functioning of the human bronchopulmonary system. One of these methods was microwave chest scanning [1], based on the fact that human tissues have a dielectric permittivity that is significantly greater than the dielectric permittivity of air. This made it possible to obtain dependences with the experimental device of changes in the levels of microwave radiation passing through various parts of the chest during respiration, called pulmonograms. However, the interpretation of the obtained pulmonograms presented certain difficulties, which led to the need to look for ways to simplify the interpretation of the results obtained by microwave sensing. 

The purpose of this article is to solve the problem of converting pulmonograms into dynamically changing images of the filling process of various parts of the human lungs with air. To achieve this goal, the following tasks were set:

(1) carrying out a series of experiments on the microwave scanning device and obtaining the results, namely pulmonograms;
(2) preprocessing of pulmonograms to increase their informativeness by a noncoherent integration of the data over several breathing cycles;
(3) development of an algorithm for converting pulmonograms into dynamically changing images;
(4) software implementation of the algorithm and imaging of the results.

## 2. Materials and Methods

### 2.1. Model for Medium and the Choice of Scanning Approach 

There are two main electromagnetic methods for investigating the medium, namely obtaining information from a backscattered wave or from an attenuated “transmitted through” wave. For the elongated spheroidal lung model, it was found that the backscattered microwave energy at the frequency of 2450 MHz is less than one-tenth of the “transmitted through” component, and the backscattered microwave energy at a frequency of 915 MHz is of the same order of magnitude as the “transmitted through” component [14]. The frequency of the generator in the experiments was 1220 MHz. The choice of frequency was based on the results obtained in [15,16], which showed the frequency dependence of the attenuation of microwave radiation that passed through the lungs of healthy and asthmatic people during respiration. The question of the optimal choice of operating frequencies, considering the penetration and attenuation properties of electromagnetic waves in human tissue at different frequencies, is not considered in this paper but does require a separate study. 

**Figure 1:**
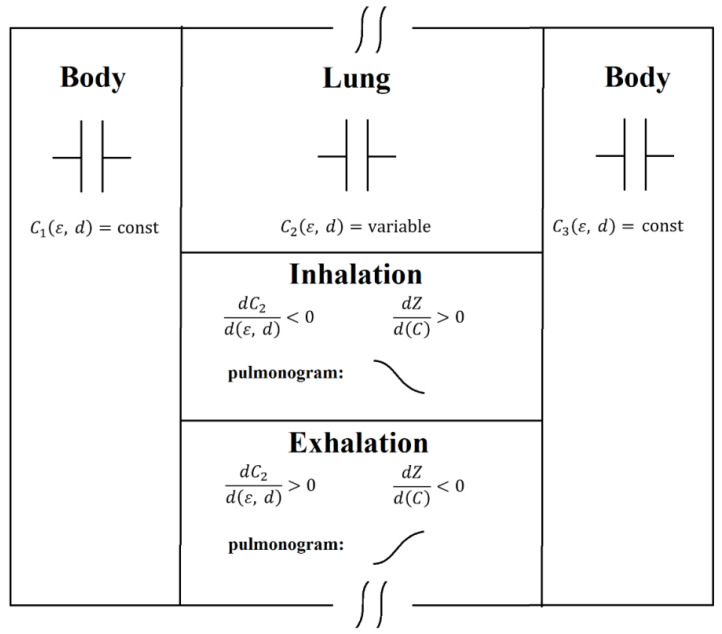
Model of the electromagnetic wave propagation medium. Breathing process is accompanied by a change in the dielectric permittivity ε and the distance d of the second capacitor, changing the impedance of the medium.

An electrical circuit consisting of three capacitors connected in series can serve as a model of the medium for an electromagnetic signal propagating through the human chest. Figure 1 shows a graphical representation of the medium model. The first capacitor simulates biological human tissues of the anterior part of the chest, in which the dielectric permittivity of the medium and the volume of these tissues do not change during respiration. The second capacitor simulates the lungs, which change their volume during respiration (the distance between the capacitor plates) and reduce the average value of the dielectric permittivity during inhalation due to an increase in the proportion of air in the lungs. The third capacitor, similar to the first one, simulates the dorsal part of the chest. Changing the amount of air in the lungs changes the reactance of the second capacitor, leaving virtually unchanged the resistance of the first and third capacitors, which changes the total impedance of the circuit of the three capacitors. In pulmonograms, air increasing in the lungs at the moment of inhalation is accompanied by a decrease in the level of the microwave signal passing through the chest compared to the signal level at the moment of total exhalation, when the amount of air in the lungs is small.

The capacitance of a parallel plate capacitor is defined as:

$$C=\frac{\varepsilon_{r}\varepsilon_{0}A}{d}$$

where $\varepsilon =\varepsilon_{r}\varepsilon_{0}$—dielectric permittivity, $\varepsilon_{0}$—permittivity of free space (constant), $\varepsilon_{r}$—relative dielectric permittivity (dimensionless), A—area of each plate, and d—distance between plates.

The relative dielectric permittivity of the air is approximately equal to 1. The relative dielectric permittivity of the lungs for the scanning frequency of 915 MHz in the air-filled condition is 21.9722, and in the absence of air, it is 51.3728 [17]. 

During inhalation, increasing the air in the lungs decreases the dielectric permittivity ε<51.3728 and increases the distance d between the plates of the second capacitor (lungs), thereby decreasing the total capacitance of the medium and increasing the total impedance of the medium:

$$Z_{total}=\frac{1}{j\omega C_{total}}=\frac{1}{j\omega C_{1}}+\frac{1}{j\omega C_{2}(\varepsilon ,d)}+\frac{1}{j\omega C_{3}}$$

During exhalation, the lungs release air, increasing the dielectric permittivity ε>21.9722 and decreasing the distance d between the plates of the second capacitor (lungs), thereby decreasing the total impedance of the medium. 

So, during inhalation, the impedance of the medium increases, which leads to a decrease in the signal level on the pulmonogram. During exhalation, the impedance of the medium decreases, which leads to an increase in the signal level on the pulmonogram. 

The chosen model is consistent with the data obtained in [17]. For the scanning frequency of 915 MHz, the lung conductivity at an air-filled condition is σ=0.4593 Sm, and in the absence of air, it is σ=0.8637 Sm. 

### 2.2. Device Block Diagram and the Principle of Its Operation

**Figure 2:**
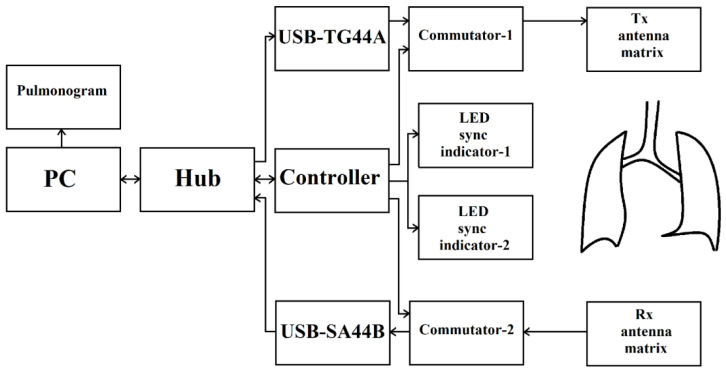
Block diagram of the device for microwave investigation of bronchopulmonary system, in particular, human respiration [2].

Figure 2 shows a block diagram of the device. The device consists of a “processing unit—PC”, an “interface unit—Hub” connected to a “microwave generator USB-TG44A” and a “Controller” that controls the operation of “Commutator-1” and “Commutator-2”, as well as “LED sync indicator-1” and “LED sync indicator-2”. Signals from “microwave generator USB-TG44A” are fed to the input of “Commutator-1”, which is connected to an array of transmitting antennas “Tx antenna matrix”. An array of receiving antennas, “Rx antenna matrix”, is connected to “Commutator-2”, the signals from which are fed into “receiver USB-SA44B” and through the “interface unit—Hub”, going to the “processing unit—PC” for processing. 

The device developed for the investigation of the bronchopulmonary system, in particular, human respiration by microwave scanning, uses a safe scanning frequency and radiation power. The technical characteristics of “microwave generator USB-TG44A” and “receiver USB-SA44B” are shown in Table 1.

**Figure 3:**
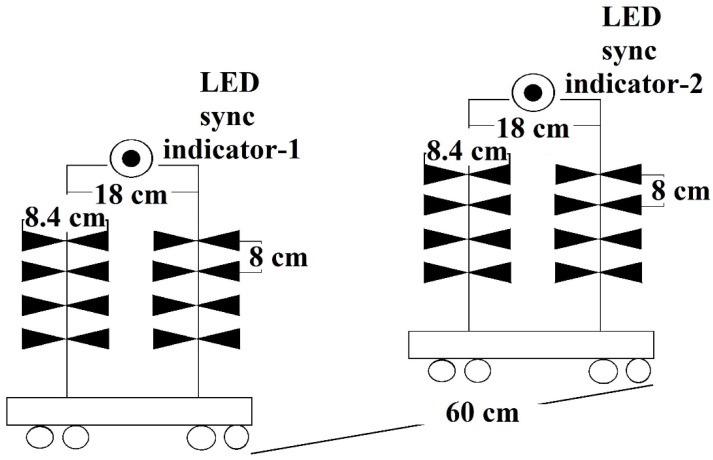
Dimensions of the array of transmitting antennas “Tx antenna matrix” and the array of receiving antennas “Rx antenna matrix” before the beginning of a series of experiments.

Figure 3 shows the dimensions of the array of transmitting antennas, namely the “Tx antenna matrix” and the array of receiving antennas “Rx antenna matrix” before the beginning of a series of experiments. The distance between the racks corresponds to the far-field propagation zone of the electromagnetic wave for the frequency of 1220 MHz. All antennas in the antenna arrays are half-wave dipoles made on FR4 laminate with a matching device providing an impedance of 50 ohms. The geometric dimensions of the antennas are shown in Figure 3.

**Figure 4:**
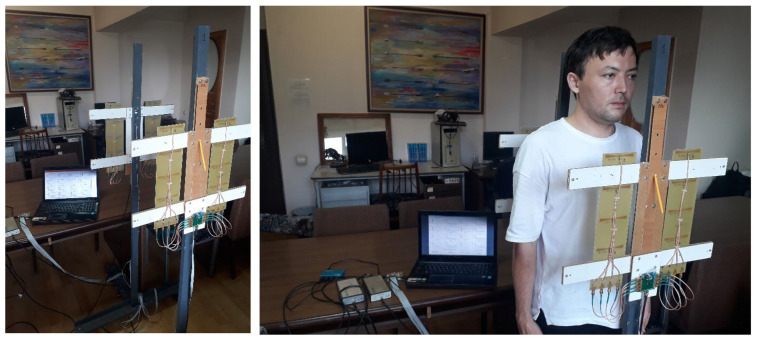
The patient examination process. Photo on the left was taken from [18].

The process of examining a patient with the microwave device to investigate the bronchopulmonary system, in particular the breathing, is shown in Figure 4 and proceeds as follows.

The examined patient stands between the racks of the array of transmitting antennas “Tx antenna matrix” and the array of receiving antennas “Rx antenna matrix”, while the matrices are installed at the level of the patient’s lungs. The LED synchronization indicators are within sight of the patient. By the operator’s command on “processing unit—PC” communication with the “Controller”, “microwave generator USB-TG44A” and “receiver USB-SA44B” is initialized via the “interface unit—Hub”. After the initialization of communication, control commands are sent to the “Controller” and “microwave generator USB-TG44A”, setting the signal power, its frequency, and the sensitivity level of “receiver USB-SA44B”. According to the signals of the “Controller”, the first and second LED synchronization indicators are turned on, in accordance with the breathing rate set by the operator and the number of breathing cycles for the procedure of obtaining pulmonograms. Guided by the LED light signals, the patient inhales and exhales. By the commands of the “Controller”, the antennas are simultaneously switched in the matrix of the transmitting antennas by means of “Commutator-1” and in the matrix of the receiving antennas by means of “Commutator-2” in order to measure different sections of the bronchopulmonary system (lung). 

The signals pass through the lungs of the examined patient, through the matrix of receiving antennas and “Commutator-2”, enter “receiver USB-SA44B”, then, through the “interface unit—Hub”, and go into the “processing unit—PC”, in which the signals received during different breathing cycles accumulate (noncoherent integration). 

The accumulated (noncoherent integration) and averaged measurement results are converted into graphs of the patient’s breathing process, which represent the pulmonograms.

### 2.3. Carrying Out Experiments

The device allows for carrying out measurements of the bronchopulmonary system of a person at calm breathing or at different rates and depths of breathing. Level and form variations in the pulmonograms can provide different information about pathological changes in the lungs and about the types of respiratory disorders, so as to identify the corresponding diseases. Coverage of the entire lung volume allows for exploring the features of the work of various parts of the lungs.

**Figure 5:**
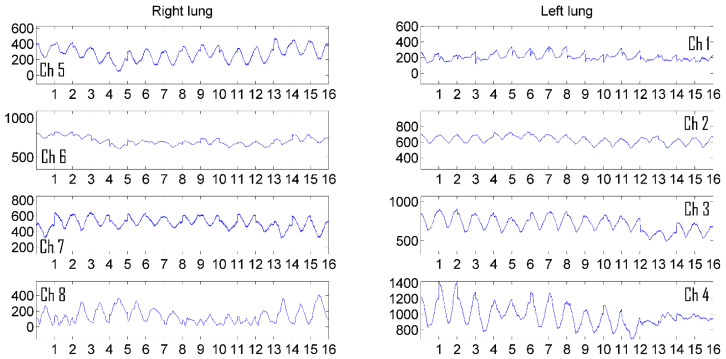
Pulmonograms of a man.

**Figure 6:**
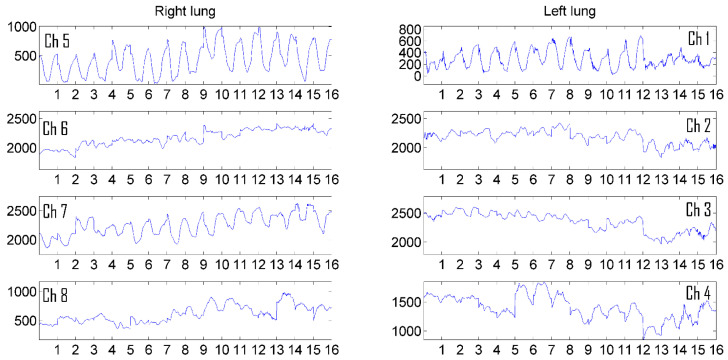
Pulmonograms of a woman.

The experiments were carried out on men and women. In total, about 20 volunteers aged 20 to 70 years were examined from among the staff of the “Granit” laboratory, in which the development of the device and the software for monitoring the functioning of the bronchopulmonary system was carried out. A series of experiments for the purpose of imaging pulmonograms was carried out at the calm breathing of patients. To demonstrate the visualization process, pulmonograms of two healthy people of different sexes are presented to demonstrate the difference in the processes of filling the lungs with air in men and women. Figure 5 shows the pulmonograms of a man, and Figure 6 shows the pulmonograms of a woman. All pulmonograms are displayed at the same scale. The number of breathing cycles is shown along the abscissa axis. Along the ordinate axis, the values are given in microvolts of the received microwave signal level. The channels of the matrix of the transmitting and receiving antennas from 1 to 4 cover the left part of the lung. The channels of the matrix of the transmitting and receiving antennas from 5 to 8 cover the right side of the lung. The upper antennas were installed at a height at the level of the clavicles. The channels are numbered from top to bottom.

Figure 5 and Figure 6 show 16 breathing cycles. Each breathing cycle is approximately 6 s (3 s for inhalation and 3 s for exhalation). Fifteen measurements were taken in each second, i.e., measurements were performed at a frequency of 15 Hz. It can be seen from the presented pulmonograms that the number of counts per breathing cycle is enough to see the temporal nature of the process.

### 2.4. Increasing the Informativeness of Pulmonograms

A significant limitation of the implementation of the method based on measuring the level of the signal transmitted through the human lungs is that the signal experiences numerous reflections inside and around the person. Minor changes in human position during measurement also affect the level of the recorded signals. Multipath propagation of the signal distorts the signal-to-noise ratio and thereby reduces the reliability, stability, and informativeness of the pulmonograms. 

To solve this problem, a method of accumulating measurement results (noncoherent integration) was proposed to increase the signal-to-noise ratio and increase the reliability, stability, and informativeness of measurement results [18]. Noncoherent integration improves the signal-to-noise ratio. Signals with an amplitude equal to 1 after M-fold summation will have an amplitude of M. The noise with a standard deviation equal to 1 before accumulation after M-fold summation will have a standard deviation of $\sqrt{M}$. Thus, the expected improvement in the signal-to-noise ratio of the voltage is: 


$$\frac{S}{N}=\frac{M}{\sqrt{M}}=\sqrt{M}$$

To implement the method of accumulation of the measurement results (noncoherent integration), it is necessary to add up the measurement results of the same duration corresponding to one complete breathing cycle. The beginning of inhalation and the end of exhalation of individual measurement results on the pulmonogram do not always coincide in duration. A person cannot exactly repeat the duration of the breathing cycle on his own. To overcome this difficulty, a method of light synchronization of human breathing cycles has been proposed. Two LEDs have been added to the device for each rack. When the LED lights up, it signals to the person about the time of the beginning of inhalation, and the turning off of the light signals to the person about the time of exhalation. Setting the time for turning on and off of the LED corresponds to the duration of a full human breathing cycle. The software allows for changing the duration of the cycles for the convenience of the patient. 

**Figure 7:**
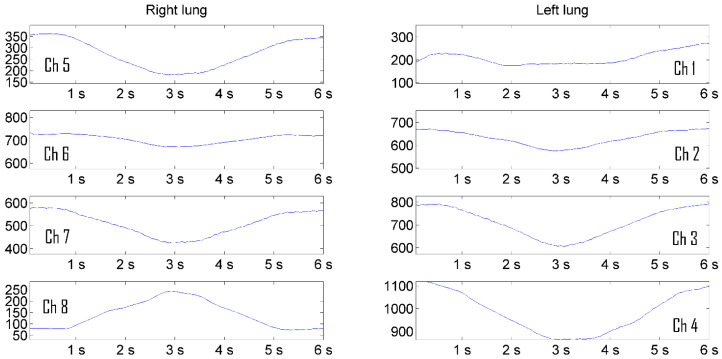
The result of accumulation and averaging of a man’s pulmonogram.

**Figure 8:**
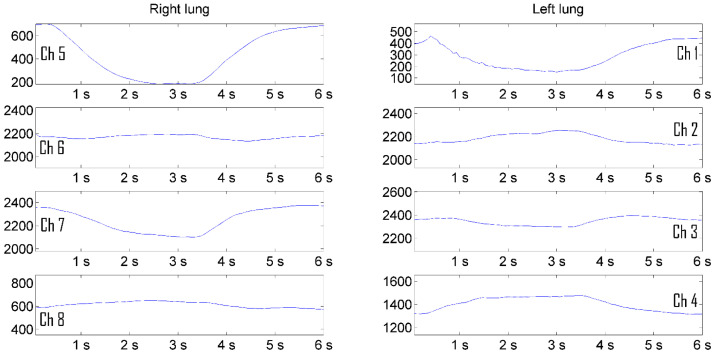
The result of accumulation and averaging of a woman’s pulmonogram.

Figure 7 and Figure 8 show the average pulmonograms of a man and a woman after noncoherent integration.

Accumulated and averaged pulmonograms are represented in a single scale, which allows for seeing the level of attenuation in different parts of the lungs and comparing them. This article presents the results of the accumulation (noncoherent integration) of 16 breathing cycles. In [19], it was mathematically proven that the necessary and sufficient number of breathing cycles for noncoherent integration is 8. The use of synchronization made it possible to effectively use noncoherent integration and thereby increase the correlation coefficient between the results, which were obtained with eight cycles of respiration, to 0.9. To further increase the informativeness of the obtained pulmonograms, an imaging algorithm described below was proposed. 

### 2.5. Imaging of Pulmonograms

**Figure 9:**
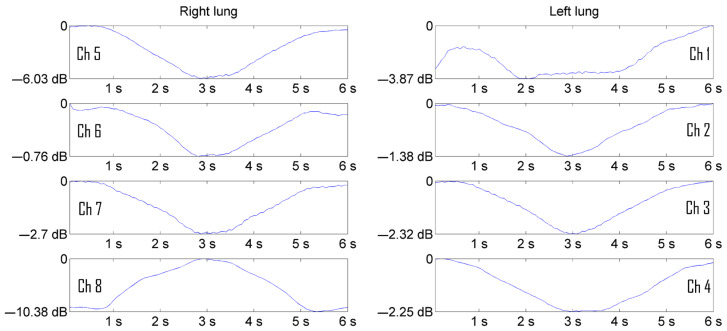
Representation of a man’s pulmonogram in decibels [18,19].

**Figure 10:**
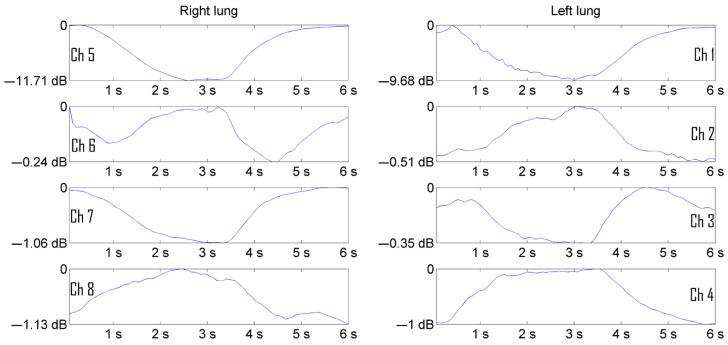
Representation of a woman’s pulmonogram in decibels.

Increasing the visibility of pulmonograms facilitates the interpretability of the measurement results. The representation of signal levels in the form of graphs turned out to be less informative and difficult for practicing pulmonologists to perceive. The solution of the visualization problem was carried out in several stages. In order for the image of the lungs not to depend on the level of the radio signal, the pulmonograms were converted into relative logarithmic units. It is more natural to represent information in relative logarithmic units. Figure 9 shows the result of presenting a man’s pulmonogram, and Figure 10 shows a woman’s pulmonogram in decibels. The maximum signal received by each antenna after the process of noncoherent integration was selected as a reference signal for conversion to decibels in each of the pulmonograms.

Pulmonograms converted into logarithmic units are the initial data for visualizing them as a color two-dimensional dynamically changing image. 

The device for measuring the functioning of the bronchopulmonary system, in particular human respiration, due to the use of electronically switched antennas in the matrix, allows it to cover the entire volume of the lungs simultaneously and measure the dynamics of air filling over time. To solve this problem, a conversion algorithm was developed, which was implemented in the Matlab language. 

**Figure 11:**
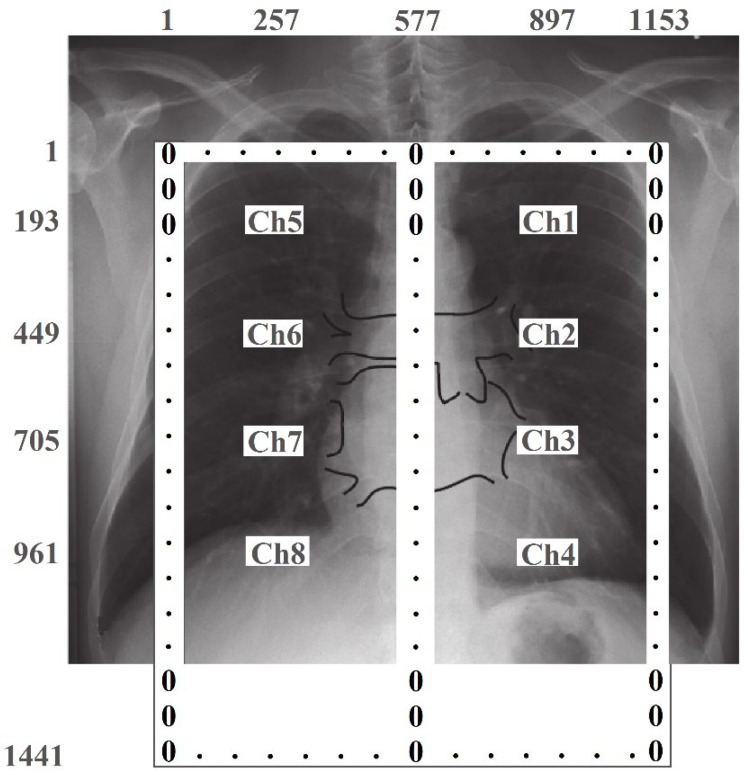
Setting the boundary values of the image matrix. The size of the display area is 1441×1153 pixels. The background image was taken from [20]. The spatial measurement points correspond to the following elements in the image matrix: Ch1 (193, 897); Ch2 (449, 897); Ch3 (705, 897); Ch4 (961, 897); Ch5 (193, 257); Ch6 (449, 257); Ch7 (705, 257), and Ch8 (961, 257).

The algorithm for converting spatially distributed pulmonograms into two-dimensional dynamically changing color images is performed in several stages. The size of the matrix for visualization was chosen as 1441×1153 pixels, which should approximately correspond to the proportions of the human chest. Since the measurements were carried out only at 8 points of the matrix, the missing elements between the 8 measurement results and the data from the a priori geometric model (boundary conditions) of the lungs were interpolated. Figure 11 schematically shows the lungs, the location of the emitter matrix, and their numbering, as well as the size of the image matrix with pixel numbering. Since, according to the a priori lung model, changes in the level of the signal associated with breathing should not occur outside and between the lungs, the edge elements of the image matrix and the elements of column 577 are assigned zero values.

At the first stage of imaging, 8 pulmonograms obtained over 16 breathing cycles were preprocessed. A noncoherent integration of the signals over 16 breathing cycles was performed. Since, at measuring pulmonograms, the time of the beginning of inhalation and exhalation was carried out according to the light signals generated by the program, the number of measurement samples in each breathing cycle is the same. To implement noncoherent integration, the pulmonogram data array was divided into 16 identical fragments, which were averaged among themselves. At the end of the first stage, 8 pulmonograms are obtained, corresponding to 8 measuring channels (Ch1...Ch8), with 1 breathing cycle in each.

In the second stage, the normalization of the pulmonograms to the maximum signal level and the logarithm of the obtained results is performed. In Matlab operators, the operations performed are written as:

**Ch1max=max(Ch1);**
**…**
**Ch8max=max(Ch8);**
**for i=1:1:n**
  $Ch1\left(i\right)=-20\log_{10}\left(\frac{Ch1\left(i\right)}{Ch1max}\right)$
  …
  $Ch8\left(i\right)=-20\log_{10}\left(\frac{Ch8\left(i\right)}{Ch8max}\right)$
**end**

In the third stage, spatial interpolation was performed. For this, the Piecewise Cubic Hermite Interpolating Polynomial (PCHIP) method was used. To perform interpolation, normalized signal values were entered into the image matrix for each breath cycle, at points with coordinates Ch1...Ch8. Interpolation fills in the missing values in columns 257 and 897. Then, the missing elements of the matrix were filled successively in all of the rows. For a sampling rate of 15 samples per second and a breathing cycle time of 6 s, 90 two-dimensional images were generated for one breathing cycle. 

In the fourth stage, the obtained two-dimensional arrays were transformed into one three-dimensional array, each layer of which (the third coordinate) corresponds to one sample of measurements. 

In the fifth stage, the display intensity scale was adjusted. To conduct this, the maximum signal (by level) was searched for in the resulting three-dimensional array, and the maximum signal on the brightness scale was equated to this value. Later, at the display of each slide corresponding to the sample time of breathing, the pixels of the slide were assigned values corresponding to the brightness scale. 

In the final stage, the slides were displayed successively at a given pace of viewing (according to sampling frequency), with the possibility of a cyclical repetition of the slides. The color scheme of the display can be changed for the convenience of the doctor.

## 3. Results 

**Figure 12:**
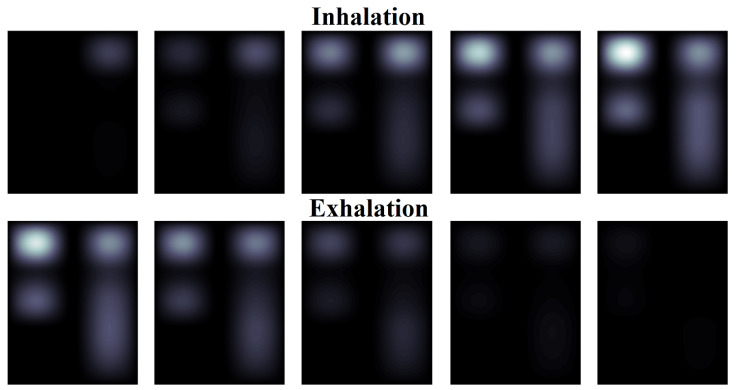
The result of imaging of a man’s pulmonograms.

**Figure 13:**
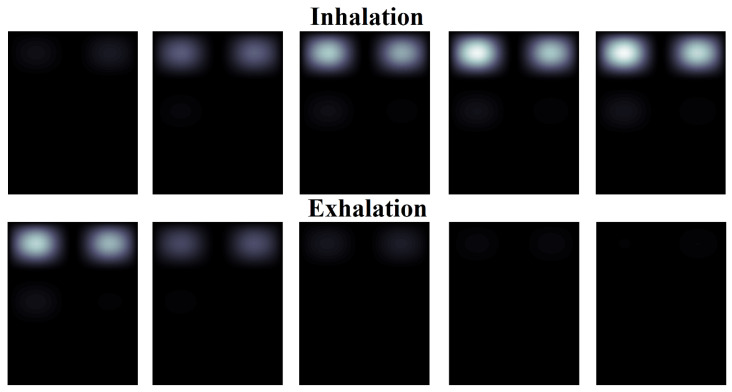
The result of imaging of a woman’s pulmonograms.

To demonstrate the implementation of the algorithm, Figure 12 and Figure 13 show the result of converting the spatial matrix of pulmonograms into dynamically changing two-dimensional color images. A gradation of one color was taken as a demonstration example. By choosing more colors to display, a comparative analysis of the filling of different sections of the lungs can be carried out. For the convenience of presenting the material, out of 90 sample slides of pulmonograms, only 10 are shown, which were obtained at regular intervals. The first 5 pulmonograms correspond to inhalation, and pulmonograms from 6 to 10 correspond to exhalation.

Figure 12 shows the imaging result for a man. The intensity of the color allows for evaluating and comparing the degree of filling of the lungs with air in different areas during breathing.

Figure 13 shows the imaging result for a woman.

An analysis of Figure 12 and Figure 13 allows for clearly demonstrating that the air occupancy of the left and right lungs is uneven in both men and women. At the same time, it can be seen that the woman who took part in the examination has a noticeable distinct thoracic breathing pattern, while the man’s lower lung is also filled, which indicates an abdominal type of breathing. At the same time, if a man’s lungs are filled with air by the middle of the inhalation period, and the air is removed by the middle of the exhalation period (uniform breathing), then the woman clearly has a faster rate of filling with air during inhalation and the same abrupt removal of air. 

The obtained results cannot be generalized to all men and women, but they show the possibility of imaging the process of filling the lungs with air even with a small number of measurement points.

Such visualization provides a more intuitive user interface for data visualization, which can help doctors more easily understand and interpret the imaging results. 

## 4. Discussion

The use of various imaging techniques in medical research largely depends on the goals of these investigations. If a diagnosis is required for the patient, then the detail of the visualized representations of the state of the organs should be as high as possible. When the question is about the preliminary diagnosis, which is typical for preventive examinations, in order to identify the early signs of the appearance of diseases, image detail is less important compared to the simplicity and cheapness of the examination procedure. 

The widespread use of software-defined radio receivers and transmitting devices makes it possible to create inexpensive devices for preventive examination. The problem of using such devices in practical medicine is the difficulty of doctors interpreting the large amount of data obtained as a result of the examination. Conversion of tabular data or graphs into two-dimensional images makes it possible to simplify the procedure for interpreting the data obtained by general diagnostic doctors who do not have special technical education, but who have knowledge of the principles of the human respiratory system. 

The proposed imaging method, according to its principle, approximates the result obtained during the examination of an X-ray image. The difference here is that the contrast agent is the air filling the lungs. Unlike the X-ray imaging method, which uses a single radiation source and a spatially distributed radiation receiver (X-ray film), the method uses eight emitters and eight radiation receivers. It would be possible to use one emitter and eight receivers while obtaining similar imaging results, but the device was designed so that, in the future, it would be possible to obtain pulmonograms between each emitter with each of the receivers. The existing device is potentially capable of obtaining not eight but 64 pulmonograms, the combination of which will allow for detailing the resulting image of lung air occupancy.

Of course, the first images of the lungs obtained using matrices of eight antennas are still quite rough. But, it is possible to estimate the potential resolution of the imaging method based on the radio frequency range used. The frequency of 1220 MHz, which was used in the experiments, corresponds to a wavelength in air (dielectric permittivity of air ≈ 1) of 24.6 cm. By passing through a human body with a lot of water (dielectric permittivity ≈ 81), the wavelength decreases by a factor of √81 = 9 and becomes 2.7 cm. Electrically conductive formations with a circumference equal to the wavelength should be good re-emitters of electromagnetic waves. This means that formations of the size π times smaller, that is, about 0.9 cm in diameter, should potentially be detected. Of course, radio waves will be scattered even from smaller inhomogeneities. But, the intensity of these scattered signals will be lower, and their images will be less contrasting.

Obviously, the use of antennas that could be attached to the human body at standard points will eliminate or significantly reduce the distortion of the results caused by the influence of human body vibrations during the examination. It will also make it possible to physiologically more accurately compare obtained results with the real geometry of the chest of the examined person. Such a technical solution will allow for reducing the effect of multipath propagation and changes in body position on the received signal.

We consider it necessary to emphasize the complete safety of the considered method in comparison with fluorography or X-ray, since the levels of microwave signals used to measure pulmonograms are many times lower than the levels recommended by ICNIRP.

Since the radio signal is attenuated not only in the lungs but also in other tissues of the body, this circumstance must be taken into account to further improve the imaging algorithms. In people with increased body weight, the signal level received by the receiving antenna should be lower than in people with normal body weight. Accordingly, in the visualization algorithm, in the future, it will be necessary to take this fact into account by introducing a correction factor to the signal level when normalizing pulmonograms.

## 5. Conclusions

The method of converting pulmonograms obtained by microwave scanning into dynamically changing color images has been proposed in the article. Despite the small number of measurement points, after the imaging of pulmonograms by the proposed algorithm, it is possible to effectively observe the filling of various parts of the lungs with air during breathing. Increasing the number of measurement points will allow for improving the resolution and detail of the imaging. 

It is shown that by using a simple, inexpensive, and safe device, it is possible to obtain images showing the process of filling different parts of the lungs with air. Taking into account that the frequency range of the radio wave radiation used in the presented experiments (1220 MHz) is even higher than the resonance frequencies of the hydrogen nuclei in MRI systems, it is possible to count on obtaining tomographic images commensurate in quality with the images of MRI devices. The difference here is that, in the MRI method, contrast is provided due to the relatively small number of hydrogen atoms in the air, and in the method under consideration, it is due to the significant difference in the dielectric permittivity of air and soft tissues of the body. The difference in physics of the examination methods is likely to result in images that differ depending upon using either MRI or RWT (radio wave tomography).

Similar results can be obtained using EIT (electrical impedance tomography). Human soft tissues conduct electric current, and the air is a dielectric. The electrical resistance of the lungs is about five times greater than that of other tissues. The difference in the dielectric permittivity of water (81) and air (1) is significantly greater, which allows us to hope for obtaining more informative images. 

The advantages of the radio wave imaging method, in comparison with MRI and EIT, can be validated and quantified based on the results of additional studies.

The obtained preliminary results made it possible to interest practicing pulmonologists and scientists. Currently, permission has been obtained to carry out preclinical tests using the proposed device. Further tests will be carried out at the Pulmonology Department of the Kazakh National Medical University named after S.D. Asfendiyarov.

## Figures and Tables

**Table 1:** Technical characteristics of “microwave generator USB-TG44A” and “receiver USB-SA44B”.

**USB-TG44A**	
Frequency range	from 10 Hz to 4.4 GHz
Radiating signal level	from −30 dBm to −10 dBm
**USB-SA44B**	
Frequency range	from 150 kHz to 4.4 GHz
Mean noise level for frequencies from 1 GHz to 2.6 GHz	−139 dBm
Dynamical range	from +10 dBm to mean noise level
Relative accuracy	±0.25 dB

## Data Availability

The Matlab code, which implements the imaging of pulmonograms, is available upon request from the corresponding author [21].
